# 

**Published:** 1887-04-02

**Authors:** 


					w PRICE ONE PENNY. > j ,
No. 27, Vol. II. w w
April 2nd, 1887.
Per Annum. 6s. 6d..nost frfiA.
IMI ML^I N51 Per Annum, 6s. 6d., post free.
<jiiu, lOOI. M\ jflr 111 HrA1 Monthly Parts, 6d.: post free, 7id
?'SiHtereo at the General Post Oflice )lL ^ -CIDitto per Ann., 7s. 6d., post free.
as a XcwKpaper.]

				

